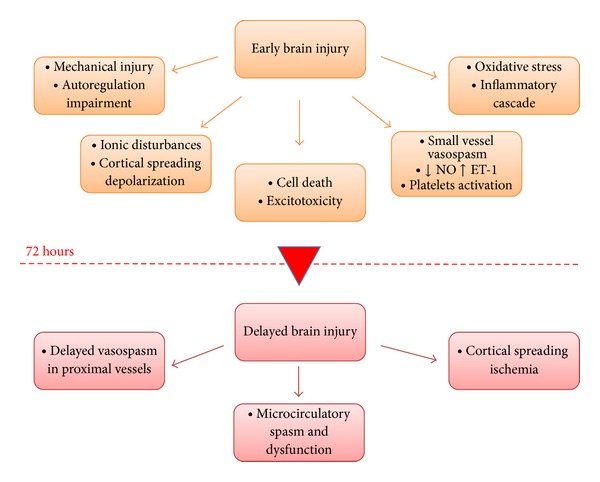# To Look Beyond Vasospasm in Aneurysmal Subarachnoid Haemorrhage

**DOI:** 10.1155/2014/628597

**Published:** 2014-05-19

**Authors:** Giulia Cossu, Mahmoud Messerer, Mauro Oddo, Roy Thomas Daniel

**Affiliations:** ^1^Department of Neurosurgery, Centre Hospitalier Universitaire Vaudois, Faculty of Human Medicine and Biology, Lausanne University, rue du Bugnon 46, 1011 Lausanne, Switzerland; ^2^Department of Intensive Care Medicine, Centre Hospitalier Universitaire Vaudois, Faculty of Human Medicine and Biology, Lausanne University, rue du Bugnon 46, 1011 Lausanne, Switzerland

## Abstract

Delayed cerebral vasospasm has classically been considered the most important and treatable cause of mortality and morbidity in patients with aneurysmal subarachnoid hemorrhage (aSAH). Secondary ischemia (or delayed ischemic neurological deficit, DIND) has been shown to be the leading determinant of poor clinical outcome in patients with aSAH surviving the early phase and cerebral vasospasm has been attributed to being primarily responsible. Recently, various clinical trials aimed at treating vasospasm have produced disappointing results. DIND seems to have a multifactorial etiology and vasospasm may simply represent one contributing factor and not the major determinant. Increasing evidence shows that a series of early secondary cerebral insults may occur following aneurysm rupture (the so-called *early brain injury*). This further aggravates the initial insult and actually determines the functional outcome. A better understanding of these mechanisms and their prevention in the very early phase is needed to improve the prognosis. The aim of this review is to summarize the existing literature on this topic and so to illustrate how the presence of cerebral vasospasm may not necessarily be a prerequisite for DIND development. The various factors determining DIND that worsen functional outcome and prognosis are then discussed.

## 1. Introduction

SAH accounts for only 5% of all strokes, with an incidence of nine per 100,000 person years [1]. Half the patients are younger than 55 years and therefore SAH has a severe economic and social impact [2]. One in six patients die during the sudden onset of bleeding and those who survive may die or deteriorate owing to early aneurysmal rebleeding, secondary delayed cerebral ischemia (DCI), hydrocephalus, or medical complications. Approximately 70% of patients die or subsequently need help with the ordinary activities of daily life [3].

Secondary DCI occurs in c. 30% of all patients [4] and results in poor outcome in half of these [3]. The high risk period for DIND is between 4 and 10 days after aneurysm rupture [4]; the pathogenesis is still incompletely understood, but classically it has been attributed to cerebral vasospasm [5].

During the last century there was a wide consensus that cerebral vasospasm was the most important determinant of poor prognosis in patients with aSAH [6]. Research was mainly directed to control and prevent delayed vasospasm, often with disappointing results. Indeed radiological improvement of vasospasm does not correlate clearly with changes in functional outcome [7, 8]. Over the last decade, growing experimental and clinical evidence has demonstrated that the presence of delayed vasospasm of the major cerebral vessels may indeed be a contributing factor but not necessarily the principal determinant of DCI and DIND. Indeed, cerebral infarction can also occur when vasospasm is not angiographically detected in the territorial artery [9] and poor outcome in aSAH seems to be directly dependent on infarction but independent of vasospasm [10]. There is increasing evidence that other coexisting factors may be involved in the development of DIND and their characterisation and treatment could improve the consistently poor clinical outcome in patients with aSAH.

The aim of this review is to discuss the various mechanisms contributing to the poor prognosis in patients with SAH and to redefine the role of delayed cerebral vasospasm in DIND.

## 2. Early Brain Injury

The term early brain injury (EBI) was first coined in 2004 to explain the acute pathophysiological events occurring within 72 hours of aSAH that begin minutes after bleeding commences [11, 12]. These events include cerebral autoregulation and blood-brain barrier (BBB) disruption, activation of inflammatory pathways, excitotoxicity, oxidative stress, and activation of apoptosis [13]. These are direct effects of the presence of blood in the subarachnoid space and also of transient cerebral ischemia. Brain injury is not limited to the primary site of haemorrhage; many of the mechanisms occurring with EBI contribute to the pathogenesis of delayed ischemic injury and are thus responsible for subsequent poor outcome. Hence early detection of EBI may make it possible to predict patient outcome; logically, therefore, early intervention that inhibits such changes may decrease mortality and improve overall outcome.

### 2.1. Mechanical Injury and Cerebral Autoregulation Disruption

Immediately after an aSAH a reactive constriction of the artery supplying the ruptured aneurysm occurs, thus producing a mechanical injury [14]. The consequence is an acute global ischemia leading to BBB disruption through endothelial cell death [15, 16]. Furthermore, both vasogenic and cytotoxic brain oedema may contribute by elevating intracranial pressure (ICP) and impairing cerebral blood flow (CBF).

Early elevation of ICP values is common after SAH. Two patterns of ICP elevation, namely, transitory and sustained, are described. The extent of rise in ICP is often used to predict outcome in SAH [17] and sustained ICP elevation is associated with higher mortality [18]. This phenomenon is associated with a severe reduction in CBF, cerebral perfusion pressure (CPP), and impaired cerebral autoregulation [19, 20], thus increasing mortality rates. Bederson et al. observed that CBF reduction to less than 40% of baseline in the first hour after SAH predicted 100% mortality, independent of ICP and CPP values [11].

### 2.2. Electrolyte Disturbances

Electrolyte disturbances are often observed within the first hours after SAH and they may be responsible for several mechanisms of EBI. Hyponatremia develops within 1-2 days from the initial bleed [21] and it occurs in 10%–30% of patients at admission; it is caused by a cerebral salt-wasting syndrome and inappropriate secretion of antidiuretic hormone. The treatment of hyponatremia is not easy and clinical signs may mimic DIND. Furthermore hyponatremic patients have a risk of developing delayed ischemic injury three times higher than normonatremic patients [22]. Risk factors for hyponatremia include a history of diabetes, chronic heart, hepatic failure, adrenal insufficiency, and NSAIDs or diuretic use [23].

**Figure 1:**
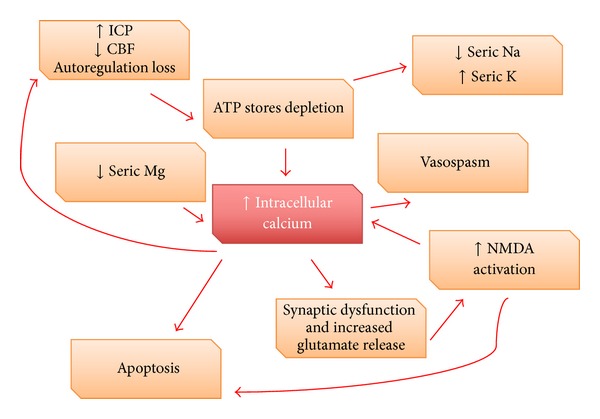


Cellular calcium homeostasis is impaired in neuronal, cerebral endothelial, and smooth muscle cells; the intracellular elevation is due to* N-methyl-D-aspartate* (NMDA) glutamate receptor activation and deregulation of adenosine triphosphatase (ATPase) dependent channels. Pathological rise in intracellular calcium may result in persistent contraction of smooth muscle cells in cerebral arteries, also causing glutamate release and activation of apoptotic pathways [24] (Figure 1).

Approximately 40% of patients admitted within 48 hours after SAH have abnormally low serum magnesium [25]. Magnesium decrease contributes to the rise in intracellular calcium by blocking NMDA receptors in an activated state and this provokes vasoconstriction, platelet aggregation, release of excitatory aminoacids, and increased synthesis of endothelin-1 (ET-1) [26].

A high level of serum potassium has been detected after SAH [27], probably owing to decreased activity in the potassium-sodium pump mechanism. Subarachnoid haemoglobin combined with a high concentration of potassium may cause widespread constriction of cerebral arteries and a pathological decrease in CBF.

### 2.3. Excitotoxicity

The increased interstitial glutamate concentration after SAH is linked to cellular leakage, altered synaptic transmission, BBB disruption, and decreased glutamate uptake [28]. In animal experiments an excitotoxicity from excessive activation of ionotropic and metabotropic glutamate NMDA receptors [29] was observed, leading to excessive intracellular calcium influx and activation of apoptotic pathways [30]. The NMDA receptor-antagonist, felbamate, improved neurological performance in rat models, limiting BBB disruption [31] and development of delayed vasospasm [32]. Similarly blood glutamate scavengers have been shown to improve neurological outcome in animal models, but the blockade of NMDA receptors may actually hinder neuronal survival [33]. In clinical studies glutamate elevation in cerebral interstitial fluid detected with microdialysis was predictive of ischemia [34] and the release of excitatory amino acid after SAH measured in interstitial and cerebrospinal fluid (CSF) correlated strongly with ICP elevation, secondary brain injury, and poor outcome [35].

### 2.4. Nitric Oxide Alterations and Endothelin-1 Increase

Alterations in nitric oxide (NO) pathways are described in the early period after aSAH both in animals and humans. [36, 37] NO is produced by nitric oxide synthase (NOS) which can be distinguished between endothelial (eNOS), neuronal (nNOS), and inducible NOS (iNOS). NO plays an important role in regulating vascular hemodynamic activity; it dilates vessels by blocking intracellular calcium release from the sarcoplasmic reticulum in smooth muscle cells and it inhibits platelet aggregation and leucocyte adhesion to the endothelial layer. Its alteration may disrupt autoregulation homeostasis and may be related to the pathogenesis of delayed vasospasm [37]. Animal studies demonstrate that cerebral NO level decreases within 10 min of aSAH [36] and it increases excessively after 24 hours [38]. The decreased availability of NO may be attributed to nNOS destruction and inhibition of eNOS through the presence of subarachnoid haemoglobin. A downregulation of eNOS and loss of nNOS in spastic arteries after SAH have indeed been demonstrated [39].

In clinical studies, increased cerebral NO levels are found 24 hours after aSAH and this indicates a poor prognosis [37, 40]. Inflammation activates iNOS and NO production may act as a vasodilator, in the form of peroxynitrite or as free radical itself, causing an oxidative stress in the vascular wall at the critical moment [41].

Endothelin-1 (ET-1) is the most potent endogenous activator of vasoconstriction, through the activation of calcium-dependent and independent mechanisms. The level of ET-1 increases in serum and plasma within minutes after SAH with a peak 3-4 days after injury [42]; it is physiologically produced by the endothelium, but in SAH there is an excessive release by astrocytes during the period of initial ischemia [43]. An upregulation of its receptors is equally observed in the delayed phase; ET_A_ receptor in particular is expressed predominantly on smooth muscle cells and is crucial in vasoconstriction and cell proliferation. ET-1 can produce long lasting vasoconstriction directly [44] and can induce morphological changes such as fibrosis or hyperplasia in the vascular wall [45]. Furthermore, a disequilibrium between NO and ET-1 level leads to unopposed vasoconstriction and promotes vasospasm development [46].

### 2.5. Oxidative Stress

Reactive oxygen species (ROS), principally oxygen free radicals, and reactive nitrogen species (RNS) are both linked to a number of vascular disease states. Oxidative stress plays a significant role in EBI. Animal and human studies indicate that ROS are generated early after SAH resulting in haemoglobin autooxidation and lipid peroxidation and a consequent rapid consumption of enzymatic and nonenzymatic antioxidant defence systems [47]. Such oxidative stress may be the trigger for a number of deleterious pathophysiological changes including structural alterations in endothelial cells, endothelial dysfunction and proliferation of smooth muscle cells [48], disruption of BBB, activation of the inflammatory cascade, and production of powerful local vasoconstrictors (e.g., leukotriene C4 and prostaglandin D2). [47]. The vasodilator effect of bradykinin in cerebral vessels through an inhibition by ROS further supports such a hypothesis [49]. The treatment of oxidative stress during the short effective therapeutic window that exists is difficult; injury caused by free radicals may well occur before a patient can receive effective treatment [50].

### 2.6. Inflammatory Pathways

The correlation between inflammation and the presence of blood in the subarachnoid space was established over fifty years ago. In 1955 Walton demonstrated that febrile patients with SAH have a worse final outcome than afebrile patients [51]. SAH triggers an inflammatory cascade: a systemic leucocytosis is commonly observed [52] and white cells can directly promote free radical formation, release cytokines and chemotactic factors to propagate the immunological response, and produce ET-1 and leukotrienes [53] and consume NO. Furthermore Spallone et al. have shown how leucocyte concentrations are more elevated in the CSF of patients with SAH-related ischemia when compared to controls [52]. By analogy elevated serum C-reactive protein levels on admission are known to be related to poor prognosis and the occurrence of delayed vasospasm [54].

Subarachnoid blood is a stimulant for nuclear factor *κ*-light-chain-enhancer of activated B cells (NF-*κ*B), which mediates the transcription of multiple components of the inflammatory cascade, including adhesion molecules, cytokines, and complement [55].

Tumor necrosis factor-alpha (TNF*α*) may also have a critical role in determining EBI. According to Starke et al., TNF*α* contributes to the formation and rupture of the aneurysm and inhibitors of TNF*α* may therefore be beneficial not only in preventing aneurysmal progression and rupture [56], but also in limiting the inflammatory process after subarachnoid bleeding.

Cytokines and chemokines may be implicated in the development and maintenance of neurovascular injury with an early increase at six hours and a late peak between 48 and 72 hours. Their elevation in serum, CSF, and microdialysis fluid is related to early and delayed ischemia and poor outcome [44, 57].

In particular, as shown by Muroi et al., higher serum interleukin-6 (IL-6) levels are associated with worse clinical outcome and DIND. Thus it is feasible that IL-6 levels may also be used as a marker to monitor clinical progression [58].

The serum and CSF levels of endothelial adhesion molecules (in particular E-selectin, ICAM, and VCAM-1), which are vital to the capture, rolling, transmigration, and diapedesis of leucocytes to the site of inflammation, are significantly elevated after aSAH [59]. Their increase within the first three days of haemorrhage is associated with poor outcome [60].

A quantitative correlation between the degree of inflammatory response and the prognosis in patients with SAH may therefore be possible.

### 2.7. Blood Breakdown Products

Haemoglobin may cause vasoconstriction by direct oxidative stress (as oxy-Hb or as bilirubin oxidation products (BOXes)) [61] and also by altering the balance between NO and ET-1. Oxy-Hb is a strong spasmogenic substance; it causes prolonged contraction of smooth muscle cells when applied to cerebral arteries in vivo and antagonists seem to prevent the occurrence of vasospasm [62]. It can catalyse the generation of superoxide and hydrogen peroxide, resulting in subsequent lipid peroxidation. Furthermore haemoglobin may scavenge nitric oxide, destroy nNOS, and alter eNOS functionality and it may indeed stimulate ET-1 production [63].

Bilirubin formation is maximal during the third or fourth day after SAH and BOXes reach a maximal concentration during the major vasospasm period (4–11 days). However, BOXes seem to be potentiators rather than initiators of vasospasm [64].

The role of iron in early brain injury after SAH was investigated by Lee et al. [65]; they showed how iron chelator desferroxamine halved mortality, attenuated DNA damage, and lessened induction of iron-handling proteins in experimental models [66]. Treatment was efficacious as early as the first day and by improving all outcomes significantly, supporting the contention that toxic blood metabolites are significant in early brain injury [66]. Both ferrous and ferric iron are prooxidant molecules and ROS may promote the transcription of NF-*κ*B and activator protein-1 [67], thus activating inflammatory pathways. ROS production catalyzed by free iron may also cause vasogenic oedema and increase ICP by disrupting BBB [68].

### 2.8. Small Vessel Spasm

Vascular spasm on angiographic imaging is restricted mostly to proximal large vessels and it occurs 3–7 days after SAH. However evidence from experimental studies shows that the constriction effect seen on parenchymal small vessels within the first minutes after SAH is greater than on large proximal vessels [11, 69]. Technical reasons limit the data on SAH-induced microcirculatory changes to animal studies. They demonstrate the presence of abnormal pial microcirculation with spasm of the microvasculatures, decreased blood flow and agglutination of red blood cells [70]. In the majority of patients, aSAH induces multiple vasospasm of arterioles without angiographic signs of vasospasm or increases in blood flow on evaluation with transcranial Doppler.

Uhl et al. confirmed constriction of small vessels in patients undergoing surgery within the first 72 h after aSAH [71] and they proposed that SAH is associated with a microvascular spasm primarily involving arterioles, with constriction in pial vessels and decrease in capillary density. Pennings later confirmed this finding [72]. In animal and postmortem pathological studies a disruption of the basal membrane and the endothelial layer was demonstrated [73], with morphological changes being more evident on parenchymal vessels compared to large vessels [74]. These may contribute to early clinical signs and may influence the postoperative course [71]. In particular endothelial dysfunction is considered to be one of the key factors initiating early vasoconstriction, keeping in with a decreased response to vasodilators (such as acethilcoline, bradikinine, or thrombin) which require a functional endothelium [75]. Basement membrane degradation seems to be more related to destabilization of microcirculation and increase in vascular permeability and interstitial oedema [76]. Whether these early changes in microcirculation can be used as a prognostic factor for the development of delayed proximal vasospasm remain to be proven.

### 2.9. Cortical Spreading Depolarization

**Figure 2:**
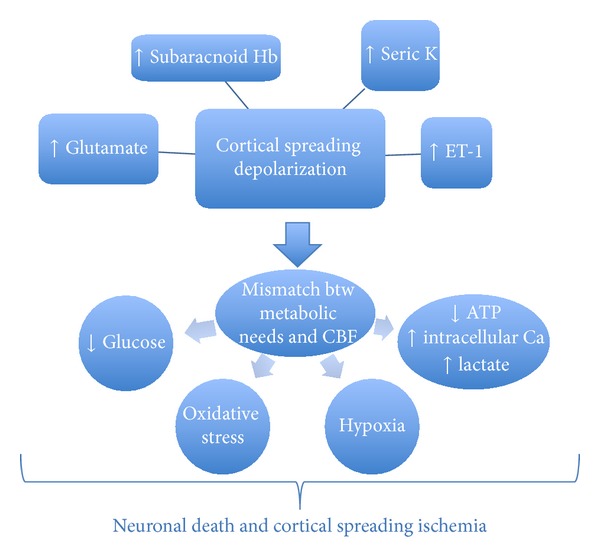


Cortical spreading depolarization (CSD) is a wave of almost complete depolarization of the neuronal and glial cells that occurs in different neurological diseases [77]. It is observed within the first 72 hours of SAH and it occurs probably as a result of the irritating activity of subarachnoid haemoglobin and an elevated extracellular potassium, glutamate, and ET-1 [78]. This results in a breakdown of ion gradients characterized by a change in the negative potential with an amplitude between −10 and −30 mV and a duration of about one minute. The histological result is neuronal oedema and dendritic distortion. The combination of decreased CBF and increased energy requirements imposed by CSD may worsen neuronal injury [79]. Clustered spreading depolarisations are related to metabolic changes suggestive of ongoing secondary damage primarily in nonischemic brain tissue [80]. Experimentally, spreading depolarization leads to massive increase in glutamate, decrease in glucose, and increase in lactate levels [81]. Under pathologic states of hypoperfusion, cortical spreading depolarization may produce oxidative stress, worsen hypoxia, and induce neuronal death [16]. Elevated intracellular calcium is possibly the predominant mediator of neuronal death from ischemia [82] (Figure 2). Clinical studies confirm how the number of spreading depolarisations recorded with a subdural electrode strip correlates significantly with the development of DCI and showed it to be a more reliable marker than vasospasm seen on angiograms [83, 84].

### 2.10. Cell Death

Secondary brain injury in particular is mediated by apoptosis, while a minor role is exercised by necrosis and autophagy. Cell death starts within 24 hours of SAH, secondary to an early decrease in CPP and CBF with the consequent activation of hypoxia-induced factors and cysteine-aspartic proteases (caspases) [85].

Serum levels of neuron specific enolase, a marker of neuronal injury, show a trend related to the amount of subarachnoid blood, which correlates with poor neurological status on admission [86]. Apoptosis is triggered by elevated ICP, ischemia, reperfusion, and acute vasospasm and by the neurotoxicity of blood breakdown components and oxidative stress [11, 44]. It involves neuronal, glial [87], and smooth muscle and endothelial cells, causing BBB disruption [88] and promoting vasospasm development. A pathological elevation of intracellular calcium activates caspase-dependent apoptotic pathways [89] and beneficial effects have been observed upon inhibition of caspase activity [90] in terms of improvement of cerebral vasospasm in animal models [91]. Furthermore after an interaction between apoptosis and autophagy was demonstrated, rapamycin and simvastatin were shown to inhibit apoptosis by activating post-SAH autophagy [92].

## 3. Delayed Brain Injury 

Many patients survive EBI but deteriorate a few days later after the hemorrhagic onset. The term delayed brain injury (DBI) describes critical events arising in the late phase of aSAH (3–14 days) resulting from the interaction of multiple pathological pathways as a direct consequence of EBI and leading to delayed cerebral ischemia (DCI) [93]. DCI causes poor outcome or death in up to 30% of patients who survive the initial impact of SAH after having their aneurysm treated effectively [94]. DCI is actually thought to be caused by the combined effects of delayed vasospasm, arteriolar constriction, thrombosis and dysfunction in microcirculation, and cortical spreading ischemia, all processes triggered by EBI.

### 3.1. Delayed Cerebral Vasospasm

Historically delayed spasm in cerebral proximal vessels was thought to be the principal factor responsible for tissue infarction and clinical deterioration and its monitoring was considered a reliable marker in the followup of aSAH patients. Several studies found a correlation between radiologically confirmed vasospasm and clinical symptoms of DCI [95]. In the acute phase it is considered the result of a prolonged contraction of smooth muscle cells, with an abnormal endothelial hypertrophy arising from inflammatory changes and gene expression modification [96]. An increase in inflammatory cells in the adventitia is observed with a necrosis in the muscular layer. In the chronic phase, a proliferation of smooth muscle cells is characteristic, probably mediated by ET-1 [97], finally leading to cerebral ischemia. Vasospasm begins on the third day after the onset of SAH with a peak at 6–8 days, eventually lasting 2-3 weeks [98]. Clinical predictors are volume, density, and prolonged presence of SAH (Fisher classification) [99] and the incidence increases with age and cigarette smoking, preexisting hypertension, and hypovolemia.

A significant relationship between severity of vasospasm and the proportion of patients with infarction was shown by Crowley et al. [100] analyzing the CONSCIOUS-1 data; a strong association was demonstrated between vasospasm seen on angiograms and new cerebral infarctions [101].

**Figure 3:**
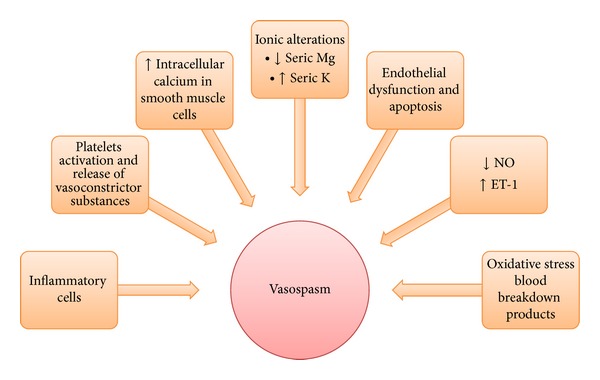


The physiopathology of delayed cerebral vasospasm is still poorly understood but many mechanisms are shared with EBI, with activation of inflammatory pathways, oxidative stress, electrolyte changes, and apoptosis activation playing an important role. Vasospasm may critically in fact be a* late* sign of EBI [102] (Figure 3).

### 3.2. Microcirculation Dysfunction and Vasospasm

Microcirculatory dysfunction is a process distinct from proximal vessel spasm. Normally autoregulation compensates for decreased CPP with a proportional vasodilatation [103]. SAH causes failure of the microcirculation, decrease in the mass density of capillaries and spasm, vasoconstriction, and pathologic changes in small vessels that may lead to infarction. Arteriolar diameter is physiologically the primary determinant for CBF and DIND is likely to be strongly related to microcirculatory changes [9].

Furthermore after aSAH, the coagulation cascade is strongly activated with a diffuse formation of microthrombi. The concentrations of fibrinopeptide A, tissue factor, and thrombin-antithrombin complexes are significantly elevated in patients who developed DCI [104]. The pathological formation of microthrombi blocking the possibility of collateral revascularisaton or causing a persistent “no-reflow” phenomenon may represent an alternative explanation for CBF reduction and DIND, independent of CPP alteration [74].

Using the index of brain tissue oxygen pressure reactivity (ORx, a variable correlation coefficient between cerebral perfusion pressure and partial pressure of brain tissue oxygen), Jaeger et al. showed how impaired autoregulation was associated with an unfavourable outcome in patients with SAH, measured according to their Glasgow Coma Score [105]. Disrupted autoregulation may predict which patients will finally develop delayed infarction [106].

### 3.3. Cortical Spreading Ischemia

Cortical spreading ischemia is a direct consequence of neuronal/glial depolarization (cerebral spreading depolarization), normally occurring 72 hours after initial haemorrhage. The direct consequence of CSD is consumption of ATP stores, electrolyte imbalance, cerebral oedema, and neuronal death, as a result of a prolonged disproportion between increased metabolic needs and decreased CBF, thus ultimately producing widespread cortical necrosis [107].

## 4. Therapeutic Strategies 

Therapeutic strategies in aSAH are currently designed to treat vasospasm with the ultimate goal of preventing delayed ischemic injury and improving clinical outcome.

### 4.1. Triple-H Therapy

Triple-H therapy (hypertension, hypervolemia, and hemodilution) was routinely used for prophylaxis and treatment of cerebral vasospasm. Hyperdynamic therapy by increasing blood pressure and, if necessary, cardiac output is considered the best available medical option for treatment of cerebral vasospasm [108, 109]. Raabe et al. showed how moderate perfusion pressure, with a CPP of 80–120 mmHg in a normovolemic hemodiluted patient, is an effective method of improving cerebral autoregulation and is associated with a lower complication rate compared with hypervolemia or aggressive hypertension therapy [110]. Similarly, Muench et al. showed in experimental models how triple-H therapy failed to improve regional blood flow more than maintaining hypertension alone. They showed that the triple-H therapy was characterized by a detrimental effect of hypervolemia and/or hemodilution which reversed the positive effect of induced hypertension on brain tissue oxygenation [111]. In conclusion, hypervolemia and hemodilution are not beneficial on CBF and are not recommended nowadays [112].

### 4.2. Calcium Channel Blockers

Current SAH treatment protocols include, besides neurointensive care and hyperdynamic therapy, the prophylactic administration of nimodipine. The rationale for the use of calcium antagonists for prevention of secondary ischemia was initially based on the blocking of the dihydropyridine-type calcium channel, thereby preventing the influx of calcium into the vascular smooth-muscle cells and decreasing the rate of cerebral vasospasm [113]. After their introduction into clinical practice it was discovered that calcium-channel blockers have neuroprotective properties and they seem to provide beneficial effects without angiographic evidence of cerebral vasodilatation [113]. Nimodipine, an L-type Ca channel blocker, is currently the only pharmacologic agent showing an improvement in neurological outcome when used for a period of 21 days after aneurysmal rupture. This occurs without a real effect on cerebral vasospasm [114]. Calcium blockers seem useful therapeutic agents outside of any effect on vasospasm [114].

### 4.3. Magnesium Sulphate

Magnesium sulphate acts as a noncompetitive antagonist of voltage-dependent calcium channels and as a NMDA-receptor antagonist and has neuroprotective and vasodilator properties [26]. Furthermore magnesium therapy seems to reduce the inflammatory burden in treated patients [115]. Intravenous magnesium sulphate was shown by van den Bergh et al. to reduce cerebral vasospasm and infarct volume after experimental SAH [25]. However, MacDonald et al. [116] and Veyna et al. [117] failed to show prevention or clinical improvement in cerebral vasospasm with magnesium therapy. It has been considered a promising agent [114] but clinical trials failed to demonstrate a clear benefit [118]. Nonetheless, the risk of adverse effects is minimal and some practitioners prefer to maintain high serum levels of magnesium in patients with aSAH.

### 4.4. ET-1 Receptor Antagonists

Disappointing results were observed in two randomized double blind phase II and III trials using an ET-1 receptor antagonist (clazosentan) [119, 120]: ET-1 seems to have a key role in vasospasm but inhibition of its action seems to reduce cerebral vasospasm without improving final functional outcome or mortality [8]. Only a small reduction was observed in the number of patients exhibiting DIND and there was no beneficial effect on the Glasgow Outcome Scale (GOS) at 3 months' followup. However the sample size estimates for CONSCIOUS-1 trial were not intended to demonstrate an effect on functional outcome and the study was underpowered to detect changes in mortality [119].

The CONSCIOUS-2 trial was designed to investigate whether clazosentan reduced vasospasm-related morbidity and all-cause mortality. It however showed no clinical benefit (including functional outcome) and systemic complications were more frequent in patients treated with this drug [120].

### 4.5. Vasodilators

Among vasodilator agents, milrinone has the added effect of being an inotrope. It is a phosphodiesterase III inhibitor that increases the level of cyclic adenosine monophosphate (cAMP); it was first used in the short-term therapy for chronic heart failure and its first use in the treatment of cerebral vasospasm after rupture of an intracranial aneurysm was reported in 2001 [121]. Furthermore it has a supposed anti-inflammatory effect [122]. However, in order to obtain a direct effect on EBI, vasodilators should be introduced immediately on hospital admission.

Recent attention was drawn to the vasodilator effects of oestrogen therapy. Ding et al. showed recently how 17 beta-estradiol (E2) is a potent vasodilator [123]. They demonstrated in vivo an attenuated cerebral vasospasm on angiography, which is probably related to a decreased iNOS expression, a normal eNOS expression, and diminished ET-1 production [124]. Furthermore, E2 may have direct antioxidant effects by scavenging ROS and it may decrease TNF*α* expression through a reduction of JNK signalling activity [125] and of inflammatory pathways. E2 may also inhibit apoptosis by neuroglobin and ERK pathway activation. However, it must be noted that known adverse effects of oestrogen treatment are not negligible.

### 4.6. Nitric Oxide Donors

NO donors were investigated in experimental studies (sodium nitrate, sodium nitroprusside, and nitrite) and they seem to prevent cerebral vasospasm in a primate model [126]. However, the clinical utility of NO is limited by its short half-life and its potential toxicity [127].

### 4.7. Antioxidants

Antioxidants such as methylprednisolone (also an anti-inflammatory agent) and tirilazad mesylate (a free radical scavenger) may prevent oxidative stress and EBI damage [128], though apparently with limited efficacy related to a one-year functional outcome [50, 129, 130]. Furthermore, free radical scavengers seem to be associated with a lower incidence of delayed ischemic injury [131].

Recently Zhang et al. [132] published a study of the use of astaxanthin (ATX), one of the most common carotenoids with potent antioxidant properties, on experimental SAH. The authors showed how ATX (by intracerebroventricular injection or oral administration) could significantly alleviate EBI in rat models by reducing brain oedema, BBB disruption, neural cells apoptosis, and neurological dysfunction. ATX may have pleiotropic effects through inhibition of glutamate release [133] by blocking inflammatory pathways (NF-*κ*B) [134], by limiting apoptosis and by platelets aggregation [135]. No side effects were reported following ATX use [136] and it may represent a new promising therapeutic option.

### 4.8. Nonsteroidal Anti-Inflammatory Agents

Different anti-inflammatory treatments have been studied in cerebral vasospasm with contrasting results. This may be explained by the heterogeneity of inflammatory patterns activated during aSAH [53]. Currently anti-inflammatory agents are not used as standard treatment in patients with SAH. However the use of nonsteroidal anti-inflammatory drugs may produce a reduction in the inflammatory response and reduce the odds for unfavourable outcomes [137].

### 4.9. Antiplatelet Agents and Inhibitors of Thrombus Formation

Clinical studies using antiplatelet agents show contrasting outcomes; one study showed a reduced risk of cerebral infarction in patients using aspirin [138] and another study showed an increased haemorrhagic volume in patients habitually using cyclooxygenase inhibitors [139].

Cerebrovascular microthrombosis was also the target of therapeutic research; ADAMTS-13 inhibits physiologically thrombus formation and thus inflammatory responses. Its systemic administration in experimental models diminished the microthrombotic process and improved neurological performances probably by limiting neuronal inflammation, without effects on vasospasm [140].

### 4.10. Statins

The debate is open for the use of statins as a therapeutic option in the acute period after SAH. Statins are hydroxymethylglutaryl- (HMG-) CoA reductase inhibitors with pleiotropic effects; they may decrease the inflammatory burden und upregulate the production of vasodilator substances (NO) by modulating eNOS expression [141, 142]. Furthermore statins may reduce the excitotoxicity of glutamate, inhibit platelet aggregation, and prevent apoptosis [143]. Some studies showed a decreased incidence of cerebral vasospasm and of mortality rate in patients treated with statins [144, 145]. One meta-analysis showed how statin use decreases the overall incidence of delayed vasospasm, ischemic injury, and mortality [146], while another meta-analysis showed no differences in outcome [147].

## 5. Discussion 

Delayed cerebral vasospasm of proximal cerebral vessels has classically been considered the primary marker to monitor patients' progression [148] and the most important and treatable cause of mortality and morbidity in SAH [149]. In the last few years, the key role of delayed cerebral vasospasm has been questioned; DIND, the principal prognostic determinant in patients surviving the acute phase, has been shown to be a multifactorial process. Multiple mechanisms other than vasospasm may contribute to long-term outcome and the role of events occurring during the immediate hours after bleeding has recently been emphasized (Figure 4).

According to the literature, 21% of aSAH survivors, who do not develop vasospasm, develop delayed ischemic injury and only 20%–30% of those who develop delayed vasospasm suffer from delayed ischemic injury [150]. The cerebral blood flow diminution observed in patients with moderate and even severe vasospasm seems, in fact, not a sufficient cause for cerebral infarction [9]. Some authors claim that many factors determine whether infarction develops after vasospasm confirmed angiographically, including the duration and severity of ischemia, the presence and length of stenosis, and the presence of collateral pathways [151]. In some cases, however, infarction occurs immediately after SAH without detectable vasospasm in the territorial artery [152] and other effects such as microthrombus formation or spasm and dysfunction in the microcirculation may make a significant contribution [153].

Therapeutic strategies in SAH patients are currently still designed to treat vasospasm with the ultimate goal of preventing delayed ischemic injury and improving clinical outcome. These therapies result either in a reduced incidence of radiologically evident vasospasm without improvement in delayed ischemic injury or in quality of life (as in the case of ET-1 receptor antagonists) or in clinical benefit without evident angiographic response in terms of decreased vasospasm (as in the case of calcium blockers).

Different reasons may be put forward to explain these results; bias in the construction of studies could play a role (e.g., sample populations being not big enough to show a real clinical benefit or low sensibility of scores chosen to evaluate clinical outcomes). Furthermore different pathological pathways may be implicated in the final outcome in patients suffering from aSAH, independent of any vasospasm demonstrated angiographically.

The role of vasospasm has probably been misinterpreted; treating vasospasm alone probably targets the wrong focus and may not lead to improvement in functional outcome. The events occurring early after haemorrhage are clearly responsible for the development of delayed ischemia; the massive brain damage observed at autopsy in patients dying within the first 72 hours of haemorrhage confirms the importance of EBI [154]. Acute intracranial circulatory arrest [18] promotes metabolic deregulation and impairment of vascular reactivity resulting in an altered autoregulation and CO_2_ responsiveness [155]. Cortical spreading depolarization, inflammation, and oxidative stress may contribute further to small vessel dysfunction, microthrombosis, and early ischemic signs [156].

Understanding, monitoring, and treating the various mechanisms at the root of early brain injury will be the key to improve the prognosis in SAH. Whilst human data are actually scant, preclinical studies demonstrate that treatment of EBI improves functional outcome.

Examining what happens early on in aSAH is usually monitored in the neurological ICU by a multimodal neuromonitoring [157]. Data existing strongly suggest that biochemical changes detected with cerebral microdialysis may precede the onset of secondary neurological deterioration following SAH [158]. Microdialysis may therefore be a useful tool to optimize neuroreanimation activities based on measures of brain metabolites in extracellular fluid, excitotoxicity and oxidative stress [40] in a the very early phase of SAH. Equally PbtO2 monitoring could help in guiding therapeutic decisions and in predicting the prognosis.

Pharmacological agents able to diminish EBI may include vasodilators (calcium blockers, magnesium sulphate, ET-1 antagonists, NO donors, milrinone, and oestrogen therapy), antioxidants, anti-inflammatory, or antiplatelet agents [13] and iron binding molecules. A combination of these therapeutic options may be necessary to obtain a synergic effect.

New promising strategies include using pleiotropic molecules with vasodilator properties (such as 17*β*–estradiol), anti-inflammatory and antioxidative drugs, such as astaxanthine or TNF*α* inhibitors, and molecules limiting microthrombus formation, such as ADAMTS-13. These showed encouraging results in preclinical studies and it is now evident that focusing on vasospasm treatment alone cannot achieve improvement in functional outcome. Promoting strategies to treat early brain injury will prevent many of the tragic consequences of SAH and new therapeutic options should concentrate further research into EBI and consequently DBI determinants [88].

## 6. Conclusions

Delayed ischemic injury is a complex process, resulting from the contribution of different pathological pathways and it is the leading determinant of poor functional outcome in patients surviving the initial hemorrhagic insult of aSAH. The role of vasospasm has long been overemphasized. Delayed vasospasm is not a necessary prerequisite for DIND development. Vasospasm alone should not be used to monitor the efficacy of therapeutic interventions nor used as a prognostic marker. Indeed, its reversal alone is inadequate as a therapeutic target. Many other mechanisms may underlie prognosis and a contemporary therapeutic approach reacting to multiple pathological pathways evident in early brain injury should be sought. Despite extensive research and aggressive management of cerebral vasospasm (both medical and endovascular), SAH prognosis remains poor. Invasive neuromonitoring to detect pathological alterations occurring in early brain injury may permit prevention of DIND; such therapeutic interventions need to be undertaken within the first hours after aSAH.

## Figures and Tables

**Figure 4:**